# Machine Learning Methods for Predicting Syncope Severity in the Emergency Department: A Retrospective Analysis

**DOI:** 10.1002/hsr2.70477

**Published:** 2025-02-23

**Authors:** Rosmeri Martínez‐Licort, Benjamín Sahelices, Isabel de la Torre, Jesús Vegas

**Affiliations:** ^1^ GCME Research Group, Department of Computer Science University of Valladolid Valladolid Spain; ^2^ Department of Signal Theory and Communications and Telematics Engineering University of Valladolid Valladolid Spain; ^3^ Department of Computer Science University of Valladolid Valladolid Spain

**Keywords:** emergency medicine, forecasting, health service administration, machine learning, syncope

## Abstract

**Background and Aims:**

Syncope is a frequent reason for hospital emergency admissions, presenting significant challenges in determining its cause and associated risks. Despite its prevalence, research on using artificial intelligence (AI) to improve patient outcomes in this context has been limited. The main objective of current study is to predict the severity of syncope cases using machine learning (ML) algorithms based on data collected during on‐site treatment and ambulance transportation.

**Methods:**

This study analyzed 572 records from five Spanish public hospitals (2018−2021), focusing on hospitalization, ICU admission, and mortality. A three‐phase strategy was used: data preprocessing, model exploration, and model selection. In the exploration phase, three data transformations techniques were applied and in each of them, models were evaluated using stratified 10‐fold cross‐validation, optimizing AUC, accuracy, and recall, with emphasis on minimizing false negatives (FN). The top‐performing models were fine‐tuned and tested. The strategy was implemented using Python libraries and a diverse set of ML classifiers were applied, including linear discriminant analysis (LDA), random forest (RF), dummy classifier (DC), and gradient boosting (GB).

**Results:**

The RF classifier performed best for predicting hospitalization, reducing FN to 37% and achieving a true negative rate (TN) of 78%, with a recall of 0.63 and accuracy of 0.74. For ICU, DC showed FN = 29%, TN = 57%, recall = 0.625, and accuracy = 0.58. The LDA classifier excelled in predicting hospital mortality, with FN = 40%, TN = 89%, recall = 0.6, and accuracy = 0.88. These results indicate that RF was superior for predicting hospitalization, while DC for ICU and LDA performed better for predicting mortality.

**Conclusions:**

This study provides an experimental foundation for the application of ML techniques in managing syncope in ED. The intention is to stimulate AI research in this area, with a view to integrating these models into clinical workflows in the future.

## 1 Introduction

Syncope is a common condition in medical practice and a leading cause of emergency department (ED) visits [1]. It manifests as a transient loss of consciousness with rapid onset and short duration, often associated with heart diseases and sudden cardiac death (SCD) [1, 2, 3]. According to recent research, syncope affects up to 30% of the population and accounts for 0.6%−3% of all ED visits [4]. Despite its small percentage, the high volume of ED visits makes syncope a significant reason for consultation. Only 50%−60% of cases receive a definitive diagnosis during the initial evaluation [4], highlighting the diagnostic challenges. Moreover, 20%−30% of patients initially diagnosed with seizures were later found to have experienced syncope [4], underscoring the complexity of managing this condition in the ED.

Syncope initial assessment in the ED involves a clinical examination to identify the underlying cause and assess prognosis [5]. However, the urgency and complexity of these cases often lead costly and unnecessary evaluations to avoid missing severe conditions [1, 3, 6]. Existing tools, such as the 2017 ACC/AHA guidelines [7], face limitations, including inconsistent definitions, lack of validation, and traditional methods that may not fully capture syncope's complexity [8, 9, 10]. For instance, the Canadian Risk of Syncope Score (CRSS) is useful for predicting 30‐day adverse events but lacks validation in diverse environments, limiting its clinical adoption [9]. Similarly, the FAINT score, though predictive, relies on specific biomarkers and suffers from sampling bias [10]. Moreover, risk assessment often requires large patient cohorts due to the rarity of adverse events [11, 12, 13, 14, 15, 16].

Current ACC/AHA guidelines emphasize the need for precise risk stratification, which is challenged by the reliance on group averages and the need for large data sets to ensure statistical significance [1]. Machine learning (ML) models, aligned with these guidelines, are trained on large data sets to uncover complex patterns not captured by traditional methods. These models provide more nuanced predictions based on a broader set of variables and iteratively adjust their parameters to optimize accuracy, thus offering more individualized predictions [17, 18, 19, 20].

ML models work by learning from labeled data (i.e., data with known outcomes) in a process known as supervised learning. During training, these models adjust their internal parameters to minimize the error between predicted and actual outcomes [6, 21]. This enables the model to better predict unseen data once training is complete. After training, the models are evaluated using a test data set to assess their ability to generalize to new, unseen data [2, 22]. These models excel at processing large data sets and identifying complex relationships between variables, which traditional methods often miss [8, 23]. This continuous learning process enhances their predictive capabilities, supporting more informed decision‐making in the ED [24]. Details on the functionality of each model are provided in Appendix SB.

Predictive algorithms for managing syncope could lead to significantly improved ED services [1], as shown by studies that have assessed the clinical risk of ED patients using ML techniques [18, 21, 22, 23, 24, 25] and artificial neural networks (ANN) [26, 27, 28, 29]. To the best of our knowledge, few studies have explored ML for syncope management in ED. These studies have generally yielded modest results and those utilizing ANN have shown modest superiority. For example, one study on short‐term adverse events achieved a sensitivity of 95% and a specificity of 67% but was prone to overfitting [26]. Another study focused on predicting hospitalization, with 100% sensitivity and 79% specificity, but faced limitations due to few input variables and lack of validation [27].

The primary objective of this research is to predict the syncope severity in ED patients arriving by ambulance, typically classified at the highest triage levels (1−3), based on prehospital assessments. The goal is to support healthcare professionals in optimizing patient management and resource allocation. To achieve this objective, a research methodology was designed and implemented. This methodology involved the creation and validation of several ML models utilizing real clinical data. The models focus on predicting three key outcomes: patient hospitalization, admission to the ICU, and in‐hospital mortality, which are indicative of serious underlying pathologies. In this way, the aim is to expand the use of ML in syncope management within the ED.

## 2 Materials and Methods

This study is a retrospective predictive analysis used ED data from five hospitals in Spanish. The research compiles with the TRIPOD checklist for presenting multivariate prediction models.

### 2.1 Data Source

The data set was compiled by a collaborating emergency physician during his clinical practice. He collected the information from patient records in the ED and compiled it into an electronic database for analysis. The original data set contains anonymized information of 572 patients with syncope treated between February 2018 and July 2021 at five hospitals in Castilla y León, Spain. The data set labels represent static categorizations based on patient outcomes recorded at the time of data collection.

The use of these data was approved as part of the project Prognostic value of the National Early Warning Score (NEWS 2) and lactic acid in the prehospital environment by the Clinical Research Ethics Committee of the hospitals involved: University Clinical Hospital of Valladolid, University Hospital of Valladolid Río Hortega, General Hospital of Segovia, University Assistance Complex of Salamanca and University Assistance Complex of Burgos. Approval ensures adherence to high ethical standards and protection of participant rights. The same ethics committees approved the collected and use of data by doctor and professor Francisco Martín Rodríguez, who acted in this research as the collaborating physician. His expertise in emergency care was applied to assist in the variable selection.

Despite coming from various centers and spanning a broad time range, the data set is limited due to the scarcity of adverse events associated with syncope in ED. The inclusion of patient records was based exclusively on a prehospital diagnosis of syncope and triage levels 1−3 (Manchester triage scale). Ensuring that only more clinically significant cases were included according to this five‐point scale, which ranges from 1 (*most urgent*) to 5 (*least urgent*). No additional selection criteria or input from physicians who treated each patient upon arrival were applied. Patients with various causes of syncope were included in the study to take advantage of the variability of the data, thus improving the learning of the ML methods. The sample size was determined by the availability in the database of patients who meet the inclusion criteria.

The original data set included 85 variables such as procedures, comorbidities, measurement of vital signs, blood gases, laboratory tests, and demographic data (see Appendix SA). Inclusion and exclusion criteria for variable selection are detailed in Table 1. The criteria allow selection compatible with the objectives of the study and its practical limitations. Practical limitations lie in the fact that this study focuses on predicting the hospital management of patients with a high level of severity. These patients may be unconscious or in a reduced state of lucidity, which makes effective communication with medical staff difficult. Sometimes, the patient will be aware of their medical history after being hospitalized.

**Table 1:** Variable selection guide for patient condition assessment.

Category	Inclusion criteria (IC)	Exclusion criteria (EC)	Variables according to the criterion (see Appendix A)
1. Patient communication	Variables that are based on objective data and do not require direct communication from the patient. (IC‐1)	Variables that depend on the patient's ability to communicate provide reliable information, such as detailed history. (EC‐1)	Comorbidities (19 variables)
2. Context and follow‐up	Variables that capture data of initial clinical relevance are useful for diagnosis and immediate treatment. (IC‐2)	Variables aimed at administrative or contextual follow‐up, such as context notes and observations not directly linked to the diagnosis. (EC‐2)	Date Days of admission Date of discharge Days in ICU Date of death Cohort description 1, 2, 3
3. Medical procedures	Variables representing essential procedures performed for the stabilization and initial diagnosis of the patient. (IC‐3)	Variables related to medical procedures performed after the initial evaluation, as they do not influence the primary classification of the patient. (EC‐3)	Diagnostic and therapeutic procedures (15 variables)
4. Demographics	Demographic variables that contribute to the analysis and statistical evaluation of the patient. (IC‐4)	—	Age Sex
5. Vital signs	Variables that represent vital signs and initial measurements upon admission. (IC‐5)	—	Measurement of vital signs (13 variables)
6. Diagnostic tests	Standard test results that assist in making immediate clinical decisions. (IC‐6)	—	Laboratory tests (20 variables)
7. Outcome data	Variables that provide information on the patient's clinical outcome. (IC‐7)	—	Outcome data (6 variables)

Initial data assessment revealed contamination issues from real‐world clinical practice. First, there is a risk of data loss when transferring patient information from the ambulance to the ED. Second, the urgency of ED decisions may result in incomplete documentation. Third, when converting analog records to digital format, errors such as loss, omission, and exchange of content between variables may occur. To address these factors, a data preprocessing step was implemented.

### 2.2 Experimental Study Strategy

**Figure 1:**
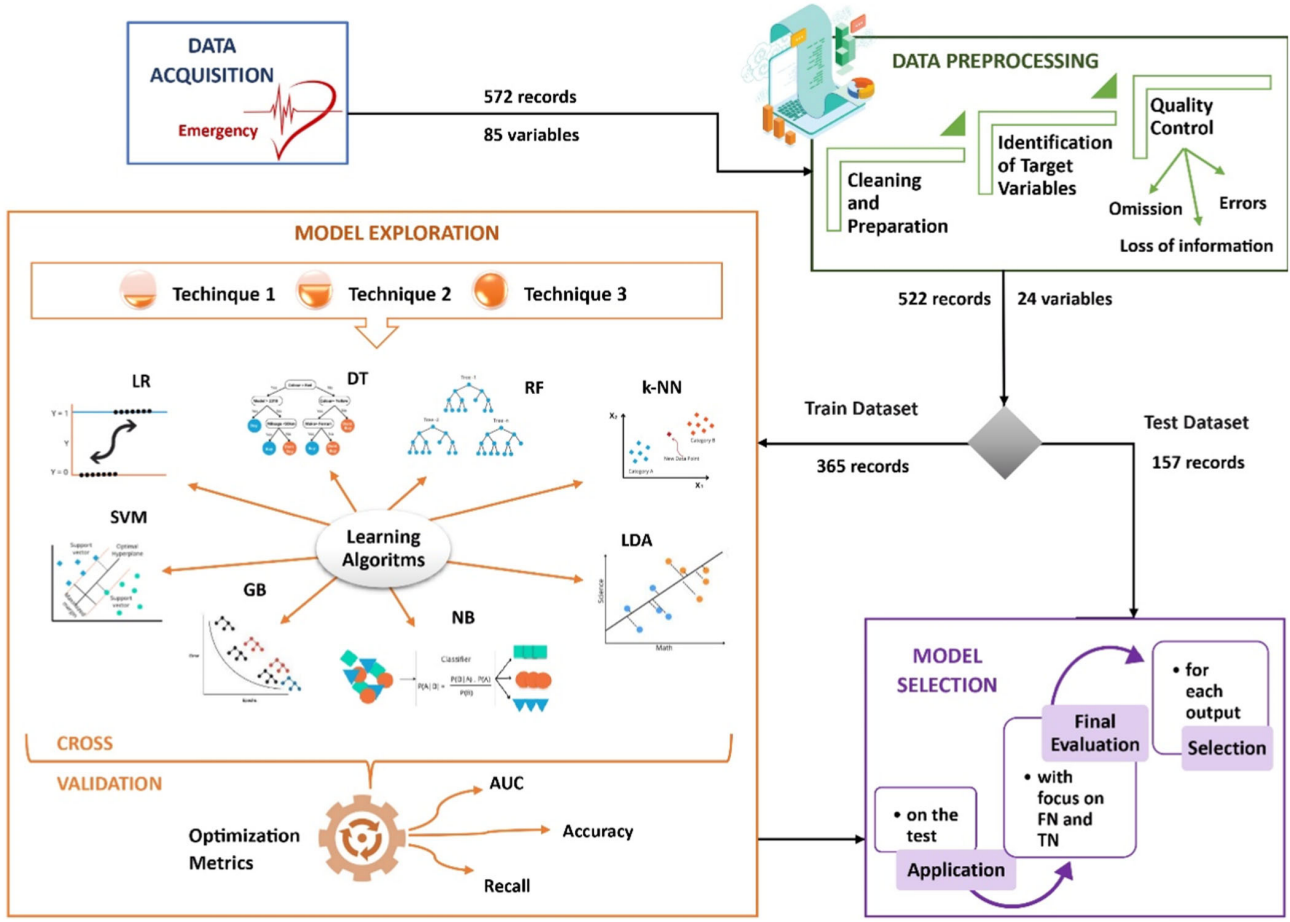
The phases of the flowchart of this research for each objective variable.

A strategy was designed whose flow diagram is shown in Figure 1, consisting of three phases: Data preprocessing, model exploration, and model selection. After data cleaning in the preprocessing phase, 70% of the data was assigned for training to the model exploration phase and 30% for testing to the model selection phase. The test set remained completely separate and unseen by the model during training, ensuring unbiased evaluation. The strategy was implemented using Python libraries including NumPy, Pandas, Tabulate, Matplotlib, Seaborn, Scikit‐learn, and PyCaret.

#### 2.2.1 Data Preprocessing

**Figure 2:**
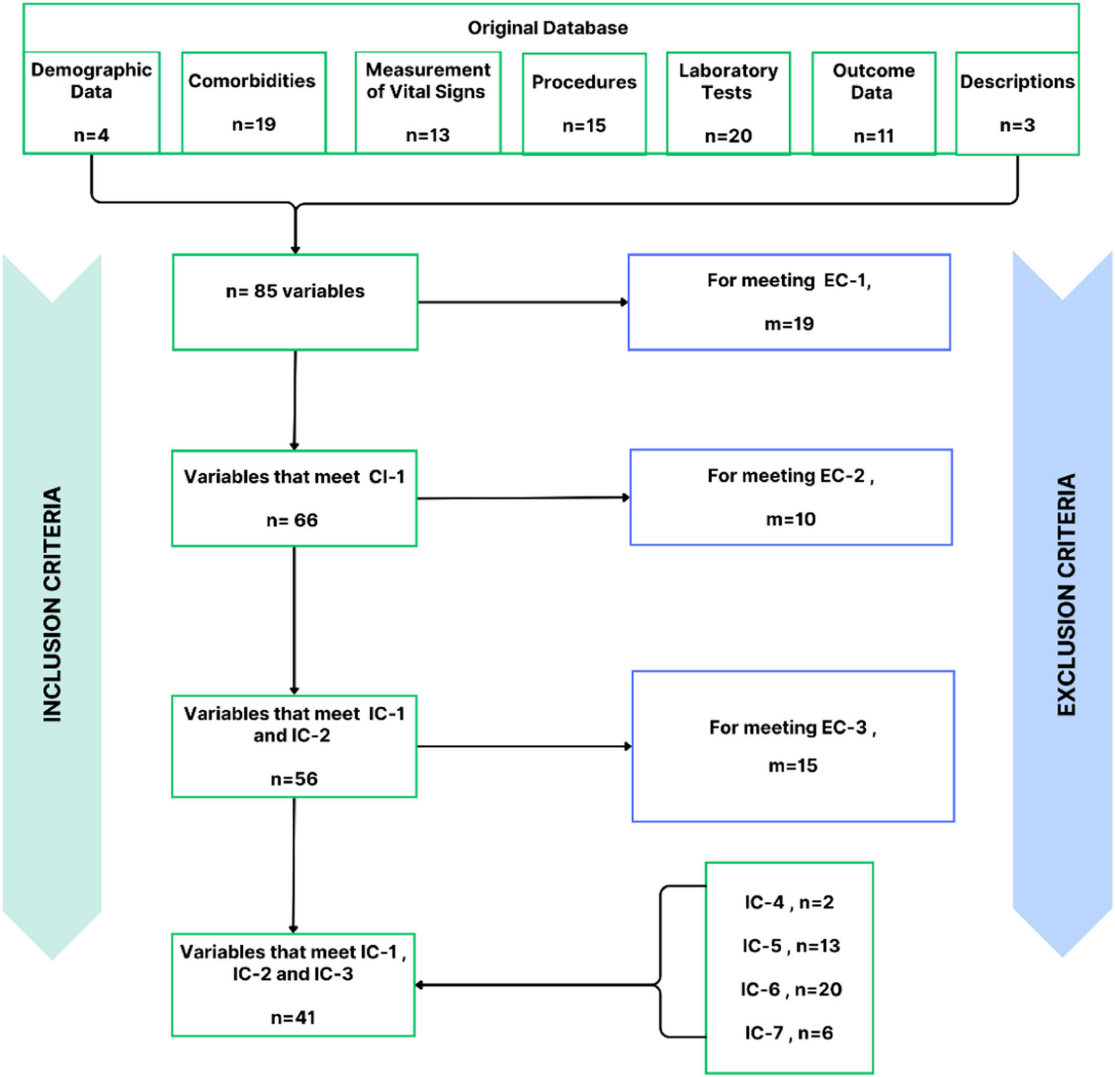
Selection of variables by inclusion and exclusion criteria.

Data preprocessing began by selecting the variables relevant to the emergency care context, aligning with the study's objectives. From the original 85 variables (detailed in Appendix SA), 41 remained after applying the inclusion and exclusion criteria described in Table 1. Figure 2 shows this process relating to the discarded variables (*m*) and the retained variables (*n*) with the respective criteria.

**Figure 3:**
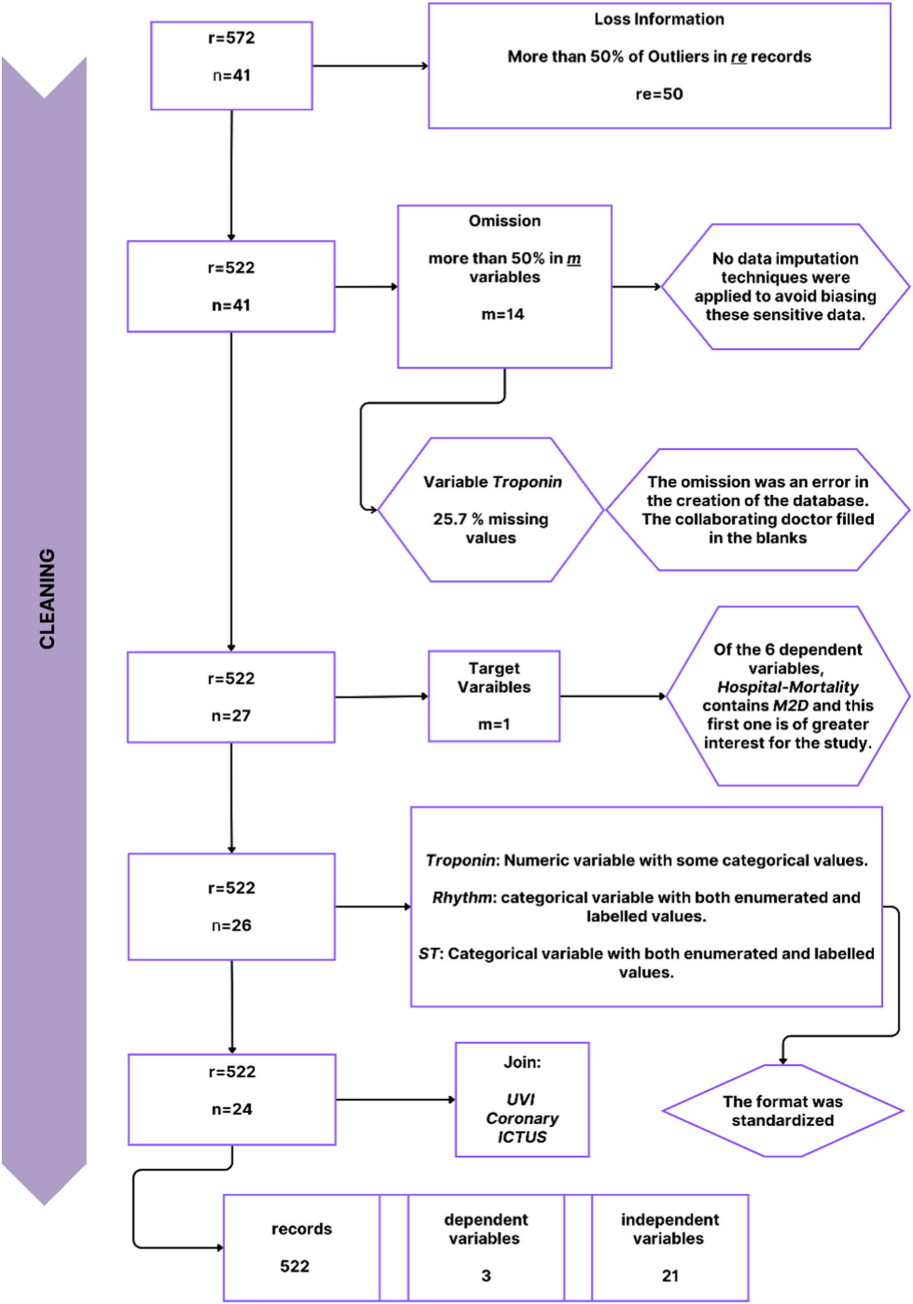
Selection of variables and records in the database cleaning process.

Figure 3 illustrates how the cleaning process affected the data set, reducing records (*r* are discarded and *re* are retained) and variables, taking the output data from Figure 2 as input. Fifty records were excluded because more than 50% outlier data, identified using a k‐NN algorithm (with a decision threshold of 0.5) and reviewed by a medical expert. In addition, 14 variables were excluded for having over 50% missing values. No imputation was applied to missing values to prevent introducing potential bias into medical data. However, the Troponin variable (25.7% missing values) was retained after the expert confirmed that the missing values could be recovered.

The M2D variable was eliminated, as its information was already included in hospital‐mortality. Likewise, errors in variable formatting were corrected, such as in Rhythm and ST, which contained categorical values sometimes identified by numbers and sometimes by names. In the case of Troponin, which had both numerical and categorical values, the numerical values were reorganized into categorical intervals in consultation with the medical expert.

#### 2.2.2 Model Exploration and Selection Framework

The model exploration phase aimed to evaluate how different levels of data transformation impact model prediction accuracy, working exclusively with the training set (70% of the original data). Based on hypotheses from technical experience and literature, three data transformation techniques were structured (see Table 2). Each incorporates transformations from the previous technique. For each transformed data set, classification methods (ML models) were applied using stratified 10‐fold cross‐validation (see Figure 1) and their performance metrics were compared. The three or four models with the best metrics were then fine‐tuned by optimizing key metrics: Area under the curve (AUC), accuracy, and recall (see Table 3). In this phase, a range of classifiers was tested to explore various modeling approaches suited to the study's objectives and data characteristics.

**Table 2:** Description of the transformations applied to the data in each model exploration technique.

**Techniques**	**Description**
First	OHE^a^
	Correction of outliers
Second	OHE
	Standardization and normalization
	Data split stratify^b^
	Weighting of classes^c^
	Collinearity removal
Third	OHE
	Selection of attributes
	Grouping of attributes
	Creation of new variables

^a^ One hot encoder. In all iterations, OHE is applied to categorical variables.

^b^ Separation of the data from the training set and the test set, depending on the class presented by each record.

^c^ In hospital‐mortality and ICU to correct the imbalance of the classes, setting both classes at 50%.

**Table 3:** Summary of classification metrics, their definitions, and best/worst values.

Metric [0−1]	Description	Best value	Worst value
True positives (TP)	Correctly identified positive cases.	High, indicates good detection.	Low, indicates poor detection.
False negatives (FN)	Positive cases incorrectly classified as negative.	Low, indicates good detection.	High, indicates poor detection.
True negatives (TN)	Correctly identified negative cases.	High, indicates good detection.	Low, indicates poor detection.
False positives (FP)	Negative cases misclassified as positives.	Low, indicates fewer errors.	High, indicates more errors.
Recall (sensitivity)	Proportion of positives correctly identified.	1 indicates complete detection.	0 indicates no detection.
Accuracy (exactitude)	Proportion of correct predictions in total.	1 indicates perfect predictions.	0 indicates erroneous predictions.
AUC (area under the ROC curve)	Ability of the model to distinguish between classes.	1 indicates perfect separation.	0.5 indicates no discrimination.

Table 4 presents two categories into which the models in this study have been divided. The first category includes those commonly used in medical literature. The second category comprises additional algorithms designed to address specific limitations of the first group, such as sensitivity to imbalanced data and difficulty capturing complex relationships between features. Table 4 summarizes the models, their key characteristics, and references to prior studies, emphasizing their relevance to the study's goals and their applicability in medical ML applications. The functionality of each model used in this study is explained in Appendix SB. This strategic combination enabled a more comprehensive evaluation, enhancing the models' robustness and generalizability. Default parameters were used due to the exploratory nature of this study and the absence of standardized configurations in the current literature.

**Table 4:** Overview of ML models used in the study: categories, key features, and literature supporting their medical applications.

Category	Model	Key features	References
Common models in medical literature	Logistic regression (LR)	Simplicity and interpretability, widely used for predicting hospitalization and mortality.	[6, 22]
	Random forest (RF)	Robustness with high‐dimensional data and nonlinear relationships, effective in emergency settings.	[21, 24]
	Gradient boosting (GB)	Iteratively improves accuracy, particularly in predicting hospital admission.	[2, 23]
	Support vector machines (SVM)	Excels in high‐dimensional spaces, with the kernel trick enabling nonlinear separation, and is applied in critical diagnostic settings.	[21, 25]
	Naive bayes (NB)	Based on probability distributions, it is simple and fast for independent features.	[8, 28]
	K‐nearest neighbors (k‐NN)	Proximity‐based classification, easy to implement, and adaptive to changes in data distributions.	[8, 28]
Additional models	Light gradient boosting machine (LightGBM)	Handles imbalanced classes effectively. Useful for predicting adverse outcomes.	[20, 24]
	Adaptive boosting (AdaBoost)	Boosting with focus on difficult‐to‐classify samples. Increases accuracy by correcting previous errors.	[20, 24]
	Linear discriminant analysis (LDA)	Assumes linear decision boundaries, reduces dimensionality, efficient with multiclass problems, and interpretable coefficients.	[4, 9]
	Quadratic discriminant analysis (QDA)	Captures nonlinear relationships, extends LDA for quadratic boundaries, handles nonlinear data effectively, and is suitable for small data sets.	[4, 9]
	Ridge classifier	Penalizes large coefficients to address multicollinearity, robust to overfitting in high‐dimensional data, and provides interpretable weights.	[17, 18]
	Extra trees (ET)	Ensemble method increases robustness to noise and reduces overfitting with randomization.	[17, 18]
	Decision trees (DT)	Simple structure, interpretable, handles both categorical and numerical data, is transparent and interpretable, and can be easily visualized	[6, 26]

AUC and accuracy were used on the training set during the exploration phase to compare models, as they provide a broad view of classification performance. A key objective was minimizing false negatives (FN), where patients requiring hospitalization or at high risk of mortality are missed. Since FN is inversely related to recall, recall was also considered a critical metric during this phase.

The model selection phase was where the models were fine‐tuned and tested on the hold‐out test set (30% of the total data). Here, the objective was to validate the generalizability of the top‐performing models from the exploration phase. These models were applied to the test set and evaluated using the metrics AUC accuracy. However, particular emphasis was placed on the confusion matrix indicators (see Table 3), specifically FN and true negative (TN). Minimizing FN ensures accurate identification of high‐risk patients while increasing TN improves resource allocation by accurately identifying low‐risk patients. A low FP minimizes misclassification. These metrics are crucial for evaluating predictive models in healthcare, as they impact clinical decision‐making and resource management. Several studies have shown that FN and TN improve the accuracy of risk stratification and prediction of outcomes like hospital admissions and mortality [1, 9, 21].

## 3 Results

### 3.1 Data Set Overview

The final preprocessed data set (see Figure 3) contained 522 records, 21 independent variables, and 3 dependent variables: Hospitalization, ICU, and hospital mortality. The ICU variable combined data from intensive surveillance units, coronary care, and stroke units to reflect severe underlying conditions. The independent variables included demographic data (age, sex), ambulance assessments (Triage, GCS), and physiological and laboratory measurements (e.g., respiratory rate, oxygen saturation, glucose, troponin).

Patients ranged from 18 to 96 years (75% over age 62). In hospital example, 75% of patients in the “nonhospitalized” group were 60 years or older, compared to 83% in the “hospitalized” group. The sample included 40.8% females and 59.2% males, which represents an 18.4% higher proportion of males than females. This difference increases in the hospitalized group, where there are 28% more males than females. These observations are noted for completeness, but they are not central to the study and should not affect the accuracy of the predictions.

A correlation analysis was conducted to explore the relationships between the variables in the data set and ensure that there were no issues of multicollinearity among the variables included in the predictive models. As expected, there was a nearly perfect correlation between GCS‐O, GCS‐V, and GCS‐M, as these represent the three components of the Glasgow coma scale (GCS). A significant linear relationship was observed between systolic (TAS) and diastolic (TAD) blood pressure measurements, with a correlation of 0.55, which aligns with medical understanding. For most variables, the correlation was weak (0.1−0.3), indicating no strong linear dependencies likely to confound outcomes. Possible causal relationships between independent and dependent variables were also explored. This analysis highlighted that critical values for some variables were common in Class 1 patients but occasionally observed in Class 0 patients as well, reflecting clinical variability.

Table 5 summarizes the distribution of classes for the three dependent variables across the entire data set following the initial data preprocessing phase before any data split. The positive class includes 137 records for the hospitalization outcome, 24 for ICU, and 15 for hospital mortality. These totals are calculated as the sum of rows where the respective classification equals “1” (e.g., hospitalization = 137 includes all rows with values of “1” in the second column). While these values may appear clinically favorable, they underscore a class imbalance, a factor considered in the modeling process. As outlined in the experimental study strategy, 70% of the data was later allocated for model training and 30% for testing, ensuring that all evaluation metrics were derived from the model's performance on the unseen test set.

**Table 5:** Distribution of patient counts by class in hospitalization, ICU, and hospital‐mortality classifications.

**Patient count**	**Hospitalization**	**ICU**	**Hospital‐mortality**
385	0	0	0
103	1	0	0
10	1	0	1
19	1	1	0
5	1	1	1
Total positive class	137	24	15

*Note:* Each row represents a unique combination of outcomes for a single patient. For instance, the first row includes patients who were not hospitalized, not admitted to the ICU, and did not experience hospital mortality (hospitalization = 0, ICU = 0, hospital‐mortality = 0). A patient may appear in more than one classification; for example, a patient who was hospitalized (hospitalization = 1) could have been admitted to the ICU (ICU admission = 1) and died (hospital‐mortality = 1).

### 3.2 Model Exploration and Selection

During the model exploration phase, models were developed using various feature selection and transformation techniques (see Table 6). The first technique focused on basic transformations (see Table 2). In the second technique, a Pearson's correlation threshold of 0.9 was set to remove highly correlated features. To approximate the normal distribution of the numerical data, the yeo‐johnson transformation method was employed. Normalization was performed using the *z‐*score method due to its higher resistance to outliers. To address class imbalance, the SMOTE estimator was implemented, performing oversampling based on the k‐NN rule. In the third technique, second‐degree polynomial features were generated to capture potential nonlinear relationships between dependent and independent variables. The SelectFromModel method from the Scikit‐learn library was used to identify and select features with importance exceeding a threshold of 0.9 in predicting the target variable. The LightGBM model (lightgbm) was chosen as the estimator for feature selection due to its speed and effective handling of imbalanced data.

**Table 6:** Parameters included in cross‐validation during model exploration.

**Techniques**	**Description**	**Value**
Second	Transformation method	Yeo‐johnson
	Normalize method	*z*‐score
	Fix imbalance methods^a^	SMOTE
	Multicollinearity threshold	0.9
Third	Feature selection method	Classic (SelectFromModel)
	Feature selection estimator	lightgbm
	Number of features selected	0.2
	Polynomial degree	2

*Note:* The first technique does not present relevant programming parameters for cross‐validation, so it is not included in the corresponding table.

^a^ Only applies to the ICU and hospital mortality classifications because they have very few samples of the positive class.

In the model exploration phase, each model was trained and evaluated using a cross‐validation with the StratifiedKFold generator. The training set included 365 patient records, representing 70% of the total data set of 522 records. The generator splits the data set into 10 folds, ensuring a similar proportion of classes in each. The model was trained 10 times, each time using onefold as the test set and the other nine as the training set, allowing for a more reliable evaluation of each model's performance. Average accuracy, AUC, and recall values were calculated for each model to identify the top performers.

After identifying candidate models, their hyperparameters were optimized using Grid Search to explore parameter combinations, selecting the one that optimized one of the three specified performance metrics. This process was applied to the best‐performing models, fine‐tuning each key metric.

In the selection phase, the optimized parameters were used to generate predictions on the test set, which included 157 patient records, representing 30% of the total data set of 522 records. The predictions were evaluated using confusion matrices and key metrics were calculated. Models that demonstrated the best balance between recall, AUC, and accuracy were selected, ensuring they were generalizable to new data. The evaluation of these models was conducted automatically, using predefined criteria and algorithms, without subjective interpretation or human intervention.

#### 3.2.1 Hospitalization

The test data analysis for hospitalization prediction revealed a class imbalance: 73.89% of the 157 observations were labeled as nonhospitalized (Class 0), while 26.11% were labeled as hospitalized (Class 1). The data showed an average patient age of 71 years, with creatinine (1.12 mg/dL) and glucose (134.06 mg/dL) levels showing relevant variability. A moderate positive correlation (0.25) between age and creatinine suggests that older patients may have higher creatinine levels, which could influence hospitalization decisions due to the associated health risks.

**Figure 4:**
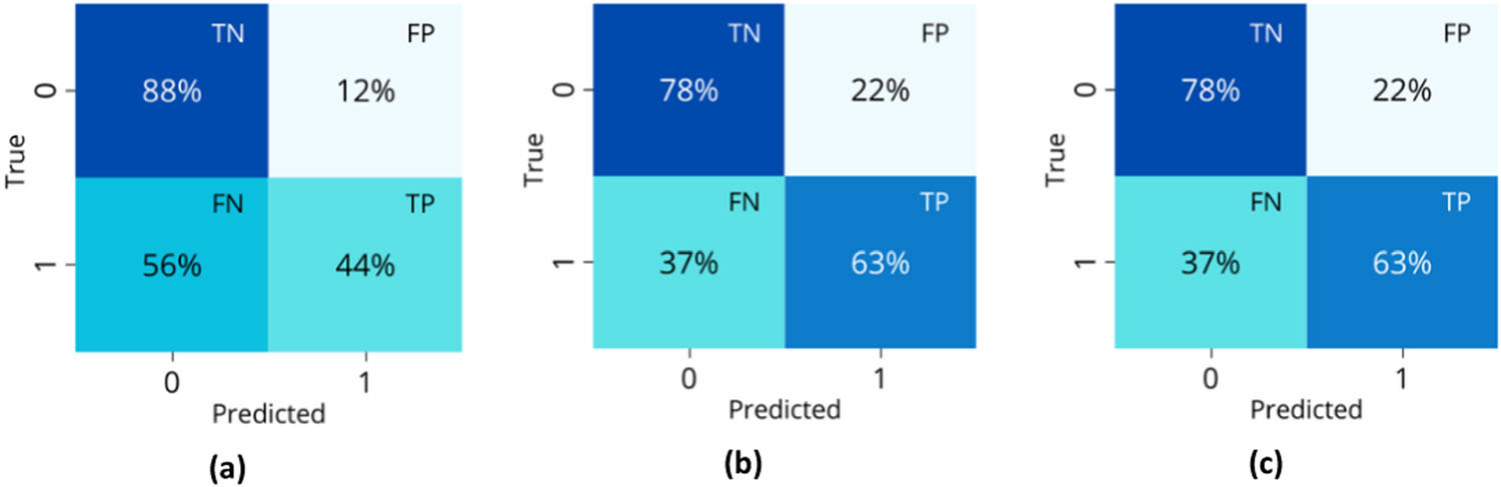
Confusion matrices of the best models for each technique of transformation in the hospitalization classification: (a) First technique with DT, (b) second technique with RF, and (c) third technique with k‐NN.

Table 7 shows the models selected during the exploration phase for each transformation technique and the reasons for their selection in the hospitalization classification. These models were evaluated in the selection phase after being optimized according to key study metrics. The best‐performing models for each of the three transformation techniques were: DT optimized for accuracy and for AUC, random forest (RF) optimized for accuracy, and k‐NN optimized for AUC, respectively. The confusion matrices for these models are presented in Figure 4. The RF under the second transformation technique was the most comprehensive model, considered the best due to its lowest FN value (37%) and overall balance in the confusion matrix, with a TN value of 78%. With a recall of 0.63 and an accuracy of 0.74, this model demonstrated robust performance in predicting hospitalization outcomes.

**Table 7:** Selection of models to evaluate hospitalization.

Models	Accuracy	AUC	Recall	Literature
First technique				
Random forest classifier	x			x
Gradient boosting classifier	x		x	
Extra trees classifier	x	x		
Decision tree classifier			x	
Second technique				
Extra trees classifier		x	x	
Random forest classifier	x	x	x	x
Light gradient boosting machine	x			
Third technique				
k‐nearest neighbors	x			
Logistic regression	x			
Random forest classifier	x	x		x

*Note:* The reasons why these models were chosen in the exploration phase are noted: to present high values in the main metrics and to be frequently mentioned in the literature consulted.

In the analysis of hospitalization results, it was concluded that while the recall metric is useful in the selection phase, it is not decisive in choosing a model during the exploration phase. Therefore, it is not included in the exploration phase tables for UCI and hospital mortality.

#### 3.2.2 ICU

The test data analysis for ICU prediction revealed a class imbalance: 95.54% of the 157 observations were labeled as not hospitalized in ICU (Class 0), while only 4.46% were labeled as hospitalized in ICU (Class 1). Descriptive statistics show that ICU patients are generally older (average age 74 years) and exhibit higher creatinine and glucose levels, indicating that these biomarkers may be linked to more severe conditions necessitating intensive care.

**Figure 5:**
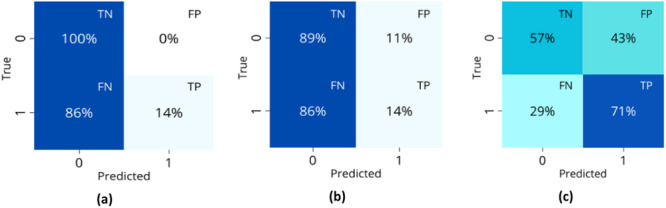
Confusion matrices of the best models for each technique of transformation in the ICU classification: (a) First technique with RF, (b) second technique with k‐NN, and (c) third technique with DC.

Table 8 details the models selected during the exploratory phase and the reasons for their choice. The best‐performing model in the selection phase was the dummy classifier (DC) optimized with AUC for the third transformation technique. Figure 5a shows this classifier's prediction, which achieved a low FN rate of 29% but a less favorable TN rate of 57%, with a recall = 0.625 and accuracy = 0.58. Subsequent techniques predominantly predicted the negative class, as depicted in Figure 5.

**Table 8:** Selection of models to evaluate ICU.

Models	Accuracy	AUC	Literature
First technique			
Random forest classifier	x	x	x
Extra trees classifier	x	x	
Light gradient boosting machine	x	x	
Second technique			
k‐nearest neighbors		x	
Random forest classifier	x	x	x
Extra trees classifier	x	x	
Third technique			
Dummy classifier	x		
Ada boost classifier		x	
Random forest classifier	x	x	x

*Note:* The reasons why these models were chosen in the exploration phase are noted: to present high values in the main metrics and to be frequently mentioned in the literature consulted.

#### 3.2.3 Hospital‐Mortality

In the analysis of test data for hospital‐mortality prediction, a significant class imbalance was observed: 96.82% of the 157 observations were labeled as negative, while only 3.18% were labeled as positive. Descriptive statistics indicated an average patient age of 70 years, with elevated levels of creatinine (mean 1.25 mg/dL) and glucose (mean 136.01 mg/dL), which may suggest increased health risks. Moderate correlations were found between hospital‐mortality and variables such as FiO2‐H (0.432) and SpO2‐H (−0.346), suggesting these factors could influence mortality risk.

**Figure 6:**
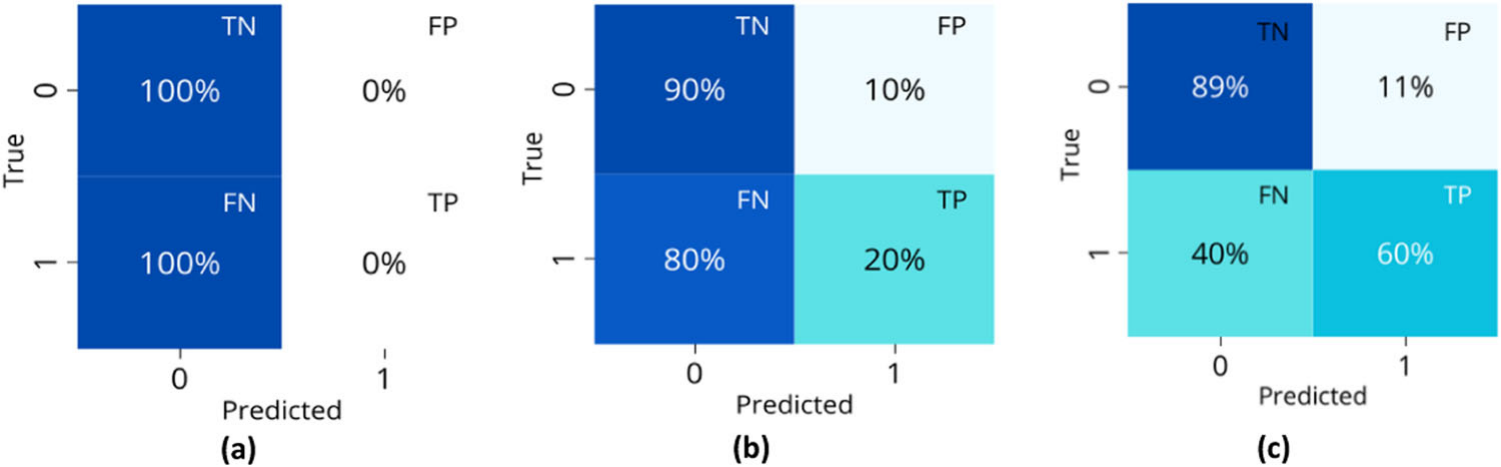
Confusion matrices of the best models for each technique of transformation in the hospital‐mortality classification: (a) First technique with RF, (b) second technique with ET, and (c) third technique with LDA.

Table 9 summarizes the models selected for hospital‐mortality classification during the exploratory phase and the rationale for their selection. In the first transformation technique, models exclusively predicted the negative class (patients with a lower likelihood of mortality), as shown in Figure 6a. The two principal models for the remaining transformation technique were k‐NN and linear discriminant analysis (LDA), with identical confusion matrices for all optimization scenarios. LDA was preferred over k‐NN due to its better performance with small, imbalanced data sets by providing clearer class separation and reducing the risk of overfitting. The LDA classifier achieved an FN rate of 40%, a TN rate of 89%, a recall of 0.6, and an accuracy of 0.88. Figure 6 shows the confusion matrices to these models.

**Table 9:** Selection of models to evaluate hospital mortality.

Models	Accuracy	AUC	Literature
First technique			
Random forest classifier	x	x	x
k‐nearest neighbors	x		
Extra tree classifier	x	x	
Second technique			
Dummy classifier	x		
Random forest classifier	x	x	x
Extra tree classifier		x	
Third technique			
Dummy classifier	x		
Light gradient boosting machine	x		
Linear discriminant analysis	x	x	

*Note:* The reasons why these models were chosen in the exploration phase are noted: to present high values in the main metrics and to be frequently mentioned in the literature consulted.

## 4 Discussion

The data in this study derived from real clinical practice and exhibited inherent variability and significant class imbalance (particularly in ICU and hospital mortality predictions). This imbalance affected model performance, often favoring the majority class (e.g., non‐ICU or low‐risk patients) and reducing sensitivity for detecting the underrepresented positive class.

For hospitalization classification, the RF model demonstrated the best balance, achieving the lowest FN among the tested models. While DT and k‐NN showed higher sensitivity to certain metrics, they struggled to achieve overall balance, often favoring the majority class. The RF model correctly predicted 78% of nonhospitalized cases while misclassifying 37% of hospitalized cases, highlighting the inherent difficulty in distinguishing patients requiring hospitalization. This challenge was exacerbated by the class imbalance and variability in key clinical predictors, which influenced the sensitivity and specificity of all evaluated models.

In ICU classification, DC was selected for its ability to minimize FN, a critical metric in identifying ICU cases, despite the significant class imbalance (95.54% non‐ICU cases). While the model showed relative strength in correctly predicting non‐ICU cases, it struggled to detect ICU cases. This imbalance impacted the model's sensitivity and specificity, reflecting the challenges of accurately predicting outcomes in underrepresented classes. Other models, such as RF and k‐NN, heavily favored the majority class, further emphasizing the difficulty of the task.

For the hospital‐mortality classification, LDA achieved a reasonable balance between sensitivity and specificity in a highly imbalanced data set (96.82% negative cases). While models like RF and ET showed high accuracy, they favored the majority class and struggled to identify high‐risk patients. LDA performed better in separating classes and reducing overfitting, but its FN rate of 40% suggests room for improvement, especially in detecting positive cases. Future work could explore reweighting strategies or ensemble methods to better handle class imbalance without compromising model sensitivity.

**Figure 7:**
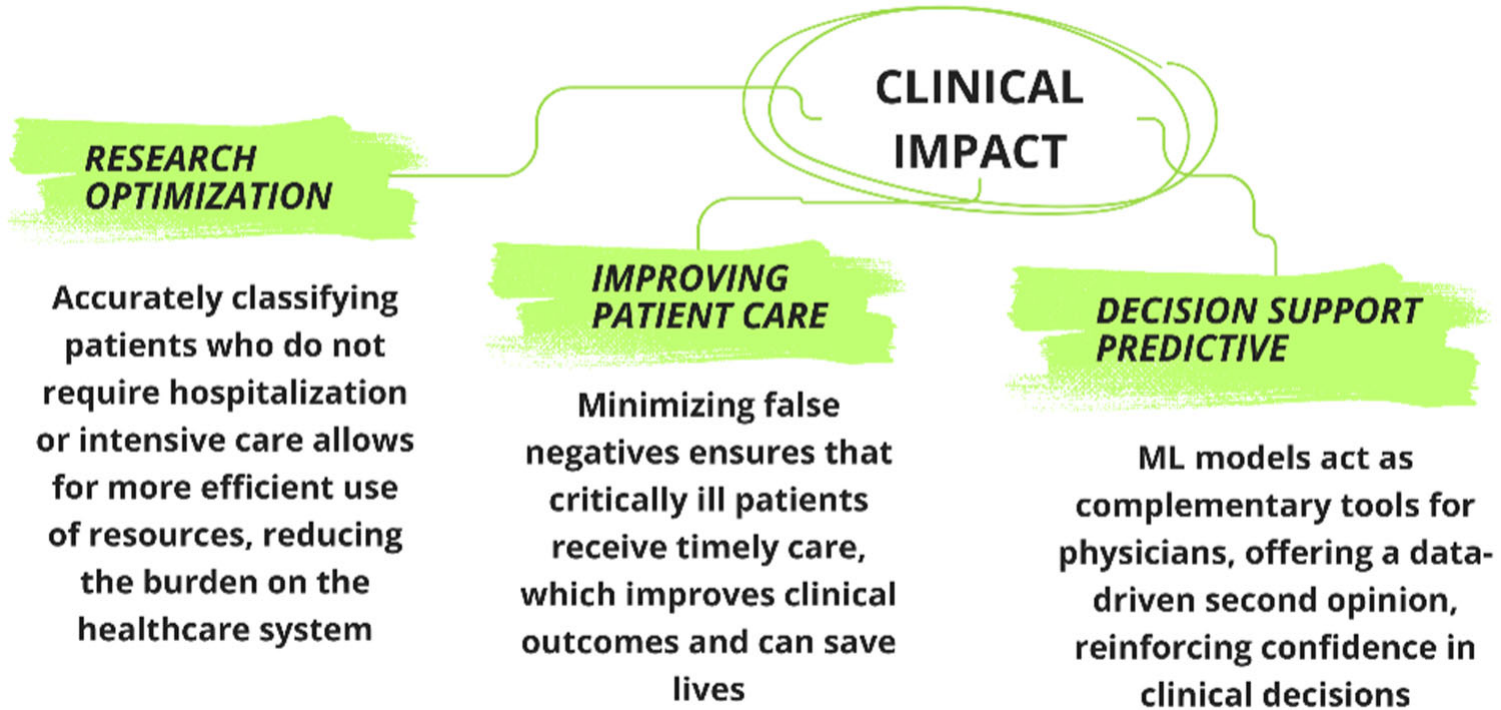
Key points of the clinical implications of ML in the management of syncope in emergencies.

This study contributes to the ML literature by demonstrating the practical application of ML in emergency care, showing its potential to enhance patient‐centered care (see Figure 7). Unlike previous studies in which cleaner data sets were used but lacked methodological transparency [26, 27]. Additionally, this study covered a broader range of models and transformations, yielding a more generalizable evaluation. Future studies should use larger data sets and apply advanced techniques like deep learning (DL) to better capture complex variable relationships and reduce data noise. Combining traditional methods with advanced techniques may also enhance the model's generalization.

These models have potential for diverse applications in emergency care. They could complement triage systems by enhancing risk assessments through prehospital data. Integration into clinical software might provide physicians with real‐time decision support during patient evaluation. Additionally, standalone tools could assist emergency staff in high‐demand scenarios. Exploring all these pathways would require stepwise validation, ensuring precision and usability in different settings, and fostering adoption by healthcare teams.

### 4.1 Limitations

Several limitations must be acknowledged. First, the small sample size may have increased the risk of overfitting, potentially limiting the models' generalizability to larger or more diverse data sets. Future research should aim to use larger, more representative data sets to improve model robustness. Data quality was compromised by intrinsic noise from clinical practice, introducing uncertainty into the results. The small sample size also restricted the ability to perform more rigorous cross‐validation. The combination of classic and novel libraries may have added unnecessary complexity, given the data set's size and nature. In future studies, simplified algorithms could improve interpretability and performance in similar contexts. Despite these limitations, efforts were made to transparently assess the findings and their implications.

## 5 Conclusions

This study developed an ML strategy to predict syncope management in the ED of public hospitals in Castilla y León, Spain, focusing on Hospitalization, ICU admission, and hospital mortality. The study identified the best‐performing models for each outcome, balancing clinical priorities (minimizing FN) and cost management (maximizing TN). The RF classifier performed best for Hospitalization (FN 37%, TN 78%), the DC excelled in predicting ICU (FN 29%, TN 57%), and LDA was most effective for hospital mortality (FN 40%, TN 89%). Compared to traditional human classification, the ML models demonstrated superior accuracy in predicting ICU and mortality outcomes. These models could help reduce unnecessary hospitalizations, allocate resources more effectively, and support clinical decision‐making. While ML models outperformed conventional approaches in accuracy, further research is needed to validate and refine these results for wider clinical application.

## Author Contributions

**Rosmeri Martínez‐Licort:** data curation, formal analysis, investigation, methodology, software, writing–original draft preparation, writing–review and editing. **Benjamín Sahelices:** conceptualization, project administration, resources, supervision, writing–review and editing. **Isabel de la Torre:** conceptualization, project administration, resources, supervision. **Jesús Vegas:** conceptualization, supervision.

## Ethics Statement

We confirm that the protocol and procedures used in this study have been and ethically approved by the Ethics Committee of the medical institutions the University Clinical Hospital of Valladolid, University Hospital of Valladolid Río Hortega, General Hospital of Segovia, University Assistance Complex of Salamanca and University Assistance Complex of Burgos. This consent has been granted in the name of the doctor and professor who collaborated with this research, Dr. Francisco Martín‐Rodríguez. This approval guarantees that the research is carried out in compliance with the highest ethical standards, thus protecting the rights and well‐being of the participants involved.

## Conflicts of Interest

The authors declare no conflicts of interest.

## Transparency Statement

The lead author, Rosmeri Martínez‐Licort, affirms that this manuscript is an honest, accurate, and transparent account of the study being reported, that no important aspects of the study have been omitted, and that any discrepancies from the study as planned (and if relevant, registered) have been explained.

## Supporting information

Supporting information.

Supporting information.

## Data Availability

The authors have nothing to report. However, they may be available upon request to the corresponding author, pending approval from the collaborating physician.
