# Supplementary material for: Machine Learning Methods for Predicting Syncope Severity in the Emergency Department: A Retrospective Analysis
Source: Health Sci Rep. 2025 Feb 23;8(2):e70477. doi: 10.1002/hsr2.70477 (PMC11847648; doi:10.1002/hsr2.70477)
Supplement: Supplementary file 1 — Supporting information. [file HSR2-8-e70477-s001.docx]

APPENDIX A. Original Variables

1. Demographic Data (4 variables)

- **Cohort**: Identifier of one of the three stages of the study (2018-2021).
- A: 2/9/2018 – 6/25/2019
- B: 11/3/2019 – 1/4/2021
- C: 4/4/2021 – 7/9/2021
- **Date**: Date of care and registration of the patient in the emergency room.
- **Age**: Age of the patient in years. Patients between 18 and 96 years old. [Continuous numerical variable]
- **Sex**: Sex of the patient. [Categorical variable: 1=Male, 2=Female]

1. Patient Comorbidities (19 variables)

*Binary variables: 1=Yes, 0=No*

- **CHF:** Chronic Heart Failure
- **AMI:** Acute Myocardial Infarction
- **PVD:** Peripheral Vascular Disease
- **CVA:** Cerebrovascular Accident
- **Hemiplegia**
- **COPD:** Chronic Obstructive Pulmonary Disease
- **DM:** Diabetes Mellitus
- **DM with injury**
- **Kidney Disease**
- **Mild Liver Disease**
- **Severe Liver Disease**
- **Ulcer**
- **AIDS:** Acquired Immunodeficiency Syndrome
- **Lymphoma**
- **Leukemia**
- **Metastasis**
- **Non-metastatic Cancer**
- **Connective Tissue Disease**
- **Dementia**

1. Measurement of Vital Signs (13 variables)

- **Triage**: Classification of emergencies. [Categorical variable: 1=vital urgency, 2=emergency, 3=urgency, 4=minor urgency, 5=non-urgent]
- **FR-H**: Respiratory rate in emergencies (breaths/minute) [Continuous numerical variable]
- **SpO2-H**: Oxygen saturation (%) [Continuous numerical variable]
- **FiO2-H**: Oxygen concentration in inspired air [Continuous numerical variable]
- **TAS-H**: Systolic blood pressure (mmHg) [Continuous numerical variable]
- **BP-H**: Diastolic blood pressure (mmHg) [Continuous numerical variable]
- **HR-H**: Heart rate (bpm) [Continuous numerical variable]
- **TT-H**: Thrombin time [Continuous numerical variable]
- **GCS.O-H**: Glasgow scale, ocular response [Categorical variable]
- **GCS.V-H**: Glasgow scale, verbal response [Continuous numerical variable] [Categorical variable]
- **GCS.M-H**: Glasgow scale, motor response [Categorical variable]
- **Rhythm**: Type of heart rhythm [Categorical variable]
- **ST:** ST segment behavior in ECG [Categorical variable]

1. Diagnostic and Therapeutic Procedures (15 variables)

- **CT**: Computerized Axial Tomography [Binary variable: Yes=1, No=0]
- **ECHO**: Ultrasound [Binary variable: Yes=1, No=0]
- **Endoscopy** [Binary variable: Yes=1, No=0]
- **Surgery**: Surgical procedures [Binary variable: Yes=1, No=0]
- **Intervention**: Non-surgical medical interventions [Binary variable: Yes=1, No=0]
- **Blood gas analysis**: Type of blood gas analysis [Categorical variable]
- **pH**: Blood pH [Continuous numerical variable]
- **pCO2**: Partial pressure of carbon dioxide (mmHg) [Continuous numerical variable]
- **pO2**: Partial pressure of oxygen (mmHg) [Continuous numerical variable]
- **cHCO3-**: Bicarbonate in blood (mmol/L) [Continuous numerical variable]
- **BE (ecf)**: Base excess in extracellular fluid (mmol/L) [Continuous numerical variable]
- **cSO2**: Oxygen saturation (%) [Continuous numerical variable]
- **TCO2**: Total carbon dioxide in blood (mmol/L) [Continuous numerical variable]
- **BE (b)**: Base excess in arterial blood (mmol/L) [Continuous numerical variable]
- **Lactate**: Lactate concentration in blood (mmol/L) [Continuous numerical variable]

1. Laboratory Tests (20 variables)

*CBC and biochemistry*

- **Hematocrit (Hct)**: Red blood cell volume (%) [Continuous numerical variable]
- **Hemoglobin (Hb)**: Hemoglobin concentration (g/dL) [Continuous Numeric Variable]
- **INR**: Prothrombin Index [Continuous Numeric Variable]
- **Leukocytes**: Total white blood cell count (10^3/μL) [Continuous Numeric Variable]
- **Platelets**: Platelet count (10^3/μL) [Continuous Numeric Variable]
- **Sodium (Na+)**: Sodium concentration (mmol/L) [Continuous Numeric Variable]
- **Potassium (K+)**: Potassium concentration (mmol/L) [Continuous Numeric Variable]
- **Glucose (Glu)**: Glucose level (mg/dL) [Continuous Numeric Variable]
- **Creatinine (Crea)**: Creatinine level (mg/dL) [Continuous Numeric Variable]
- **Bilirubin**: Bilirubin level (mg/dL) [Continuous Numeric Variable]
- **C-Reactive Protein (CRP)**: Level CRP (mg/L) [Continuous Numeric Variable]
- **Procalcitonin (PCT)**: Procalcitonin levels (ng/mL) [Continuous Numeric Variable]
- **D-Dimer (Dimer)**: D-dimer level (μg/L) [Continuous Numeric Variable]
- **proBNP:** B-type natriuretic peptide prohormone (pg/mL) [Continuous Numeric Variable]
- **Troponin (Trop)**: Troponin levels (ng/mL) [Continuous Numeric Variable]
- **Creatine Kinase (CK)**: CK level (U/L) [Continuous Numeric Variable]
- **Neutrophils**: Neutrophil count (10^3/μL) [Continuous Numeric Variable]
- **Lymphocytes**: Lymphocyte count (10^3/μL) [Continuous Numeric Variable]
- **Urea**: Urea level (mg/dL) [Continuous numerical variable]
- **LDH**: Lactate dehydrogenase level (U/L) [Continuous numerical variable]

1. Outcome Data (11 variables)

- **Hospitalization**: Hospitalization indicator [Binary variable: Yes=1, No=0]
- **Days of admission**: Days of hospital stay.
- **Date of discharge**: Date of hospital discharge.
- **UVI**: Indicator of admission to Intensive Surveillance Unit [Binary variable]
- **Coronary**: Indicator of admission to coronary care unit [Binary variable]
- **ICTUS**: Indicator of admission to stroke unit [Binary variable]
- **Days in ICU**: Days in specialized units (UVI, Coronary or STROKE).
- **Hospital mortality**: Indicator of death during stay [Binary variable]
- **Days to death**: Days from admission to death.
- **Mortality in 48 hours (M2D)**: Indicator of death in the first 48 hours [Binary variable]
- **Date of death**: Date of death.

1. Clinical Descriptions (3 variables)

*Descriptions related to diagnosis or treatment*

- **Description 1**: Initial diagnosis in the emergency room.
- **Description 2**: Hospital follow-up notes, recording complications or additional events.
- **Description 3**: Hospital follow-up notes, recording complications or additional events.
