# Supplementary material for: Machine Learning Methods for Predicting Syncope Severity in the Emergency Department: A Retrospective Analysis
Source: Health Sci Rep. 2025 Feb 23;8(2):e70477. doi: 10.1002/hsr2.70477 (PMC11847648; doi:10.1002/hsr2.70477)
Supplement: Supplementary file 2 — Supporting information. [file HSR2-8-e70477-s002.docx]

APPENDIX B. Detailed Explanation of Individual Model Functionality

1. **Logistic Regression (LR)**:

- Training:
  - Logistic Regression is a linear model used for binary classification. It works by calculating the probability that a given input belongs to a particular class.
  - During training, it estimates the weights (coefficients) for each feature using optimization algorithms like gradient descent. This process minimizes the error between predicted probabilities and actual class labels using a logistic function (sigmoid function).
- Prediction:
  - After training, the model predicts a probability that the input data point belongs to a particular class. This is done by calculating a weighted sum of the input features and passing it through the sigmoid function.
  - If the probability is greater than a threshold (usually 0.5), the data point is classified as belonging to one class; otherwise, it is classified as belonging to the other class

1. **Random Forest (RF)**

- Training:
  - Random Forest is an ensemble method that builds multiple decision trees, each trained on a random subset of the data (bootstrapping) and using a random subset of features for each tree split.
  - The algorithm then aggregates the results of individual trees (usually through voting for classification or averaging for regression) to make the final prediction. This process is known as bagging (Bootstrap Aggregating), which helps reduce overfitting.
- Prediction:
  - When a new data point is provided, each individual tree in the forest makes a prediction. For classification, the class that gets the most votes from the trees is selected as the final prediction. For regression, the predicted value is the average of all individual tree predictions.

1. **Gradient Boosting (GB)**:

- Training:
  - Gradient Boosting is an ensemble learning method where trees are built sequentially. Unlike Random Forest, where trees are built independently, in Gradient Boosting, each tree is trained to correct the errors made by the previous trees.
  - It starts with a simple model (usually a decision tree), calculates the residuals (errors), and fits a new tree to those residuals. The goal is to iteratively reduce the errors in prediction by adjusting the model with each new tree.
- Prediction:
  - Once the model is trained, predictions are made by combining the outputs of all trees. Each tree in the sequence adds a small correction to the overall prediction. The final output is a weighted sum of the predictions from all the individual trees.

1. **Support Vector Machines (SVM)**:

- Training:
  - SVM is a supervised learning algorithm that works by finding the hyperplane that best separates the data points of different classes. This separation is done in a high-dimensional feature space, which allows SVM to handle complex, non-linear decision boundaries.
  - The algorithm attempts to maximize the margin (the distance between the closest data points of each class to the hyperplane). This is done by solving an optimization problem.
- Prediction:
  - Once trained, SVM classifies new data points by determining which side of the hyperplane they fall on. The classifier assigns the label of the closest class based on the position relative to the hyperplane.

1. **Naive Bayes (NB)**:

- Training:
  - Naive Bayes is a probabilistic classifier based on Bayes' theorem. It assumes that the features are conditionally independent given the class (hence "naive").
  - During training, it calculates the probability distributions of each feature for each class. These probabilities are estimated from the training data and used to compute the likelihood of a new data point belonging to each class.
- Prediction:
  - For prediction, Naive Bayes calculates the posterior probability of each class, given the feature values of the new data point. It assigns the class with the highest posterior probability, which is computed as the product of the likelihood of each feature given the class and the prior probability of the class.

1. **K-Nearest Neighbors (K-NN)**:

The term "training" for **K-Nearest Neighbors (K-NN)** is somewhat misleading compared to other models. Unlike algorithms such as Logistic Regression or Random Forest, K-NN does not involve parameter optimization or building an explicit model during a training phase.

- Data Storage (Pseudo-Training):
  - K-NN "remembers" the entire training dataset, storing each instance (data point) with its corresponding label. This is sometimes referred to as the "training phase," but no actual model-building or parameter optimization happens in this phase. The algorithm does not generalize based on the data; it simply stores it for future comparison.
- Prediction:
  - When a new data point (test data) is provided, K-NN computes the distance between this new point and all other points in the training set. The distance is typically measured using metrics like Euclidean distance (straight-line distance), Manhattan distance (distance along axes), or Minkowski distance (a generalization of both).
  - After computing the distances, it identifies the K nearest neighbors (the closest points in the training set).
  - The model then predicts the class of the new data point based on a majority vote among the K neighbors (for classification) or by averaging their values (for regression). The algorithm "learns" from the dataset by applying the principle of similarity: the more similar a data point is to others, the more likely it is to belong to the same class or have similar outcomes.

1. **Light Gradient Boosting Machine (LightGBM)**:

- Training:
  - LightGBM is a gradient-boosting framework optimized for efficiency and speed. It uses histogram-based learning, which reduces computational overhead compared to traditional boosting methods.
  - It splits trees leaf-wise (instead of level-wise), allowing it to focus on areas of the data with the most errors, improving accuracy while preventing overfitting with proper regularization.
  - Handles large datasets and works effectively with high-dimensional and sparse data.
- Prediction:
  - Similar to other gradient boosting models, LightGBM combines the outputs of multiple sequential trees to make predictions.
  - For classification, it outputs probabilities that a data point belongs to a class, which can then be thresholded for binary decisions.

1. **Decision Trees (DT)**:

- Training:
  - Decision Trees work by splitting the dataset into subsets based on feature values. At each node, the algorithm selects the feature that best divides the data into homogeneous groups (using metrics like Gini impurity or entropy).
  - Training ends when a stopping criterion is met, such as reaching a maximum depth or having no further information gain.
- Prediction:
  - New data points are passed through the tree, following the splits determined during training, until they reach a leaf node. The class label or prediction value at that leaf is assigned to the data point.

1. **Extra Trees (ET)**:

- Training:
  - Extra Trees is an ensemble method similar to Random Forest, but with a key difference: it selects splits randomly (instead of choosing the best split based on criteria like Gini impurity or entropy).
  - This randomness makes the model less sensitive to noise and faster to train while maintaining high accuracy on large datasets.
- Prediction:
  - Like Random Forest, it aggregates predictions from all the trees in the ensemble (via majority voting for classification or averaging for regression).

1. **Linear Discriminant Analysis (LDA)**:

- Training:
  - LDA projects data into a lower-dimensional space where class separability is maximized. It assumes that the features of each class are normally distributed and have the same covariance structure.
  - It calculates the discriminant functions to find the hyperplane that best separates classes while preserving maximum variance within classes.
- Prediction:
  - LDA assigns a new data point to the class with the highest posterior probability, calculated using the learned discriminant functions.

1. **Ridge Classifier**:

- Training:
  - Ridge Classifier is a regularized linear model that minimizes a loss function penalized by the L2 norm of the weights. This regularization helps reduce overfitting, especially when dealing with multicollinearity (correlated features).
  - It solves for weights that balance model complexity with accuracy.
- Prediction:
  - Once trained, it applies a linear decision boundary to classify data, assigning labels based on which side of the boundary a data point lies.

1. **Adaptive Boosting (AdaBoost)**:

- Training:
  - AdaBoost builds a sequence of weak classifiers (typically decision stumps) and focuses on correcting the errors made by the previous classifiers.
  - It assigns weights to misclassified samples, increasing their importance in subsequent iterations. This adaptive process emphasizes difficult-to-classify cases.
  - Final predictions are a weighted sum of the predictions from all weak classifiers.
- Prediction:
  - AdaBoost aggregates the predictions from its weak learners, weighting each learner's contribution based on its accuracy.
  - The final classification is based on the aggregated result, typically using a majority vote or a threshold.

1. **Quadratic Discriminant Analysis (QDA)**:

- Training:
  - Similar to LDA, but QDA does not assume that all classes share the same covariance matrix. Each class has its own covariance structure, making it better suited for non-linearly separable data.
  - It computes a quadratic decision boundary rather than a linear one.
- Prediction:
  - QDA assigns a new data point to the class with the highest posterior probability, leveraging the quadratic boundaries for classification.
